# ‘What happens if I do nothing?’ A Systematic Review of the Inclusion and Quantitative Description of a ‘No Active Intervention’ Option in Patient Decision Aids

**DOI:** 10.1007/s11606-020-06444-3

**Published:** 2021-02-02

**Authors:** Tammy Hoffmann, Julia Beckhaus, Chris Del Mar

**Affiliations:** Institute for Evidence-Based Healthcare, Bond University, Gold Coast, Queensland Australia

## INTRODUCTION

The tendency of patients and clinicians to overestimate intervention benefits is an acknowledged contributor to healthcare overuse.^1,2^ For many conditions, not actively intervening is a legitimate option, such as for self-limiting conditions which can resolve without intervention (other than for symptom management), for conditions where ‘wait and watch’ is appropriate, or where not having a screen or test is reasonable. As part of shared decision-making and campaigns such as Choosing Wisely,^3^ patients are encouraged to ask ‘what happens if I do nothing?’. To help patients construct informed preferences and decisions congruent with their values, part of a decision aid’s role can be to correct misperceptions about the benefits and harms of intervention options, including not intervening.^4^ For conditions where not actively intervening is reasonable, informed decision-making requires discussion about the condition’s natural history, such as timeframes to recovery or other likely consequences. We aimed to analyze the inclusion and quantitative description of a ‘no active intervention’ option in all publicly available decision aids.

## METHODS

**Figure 1:**
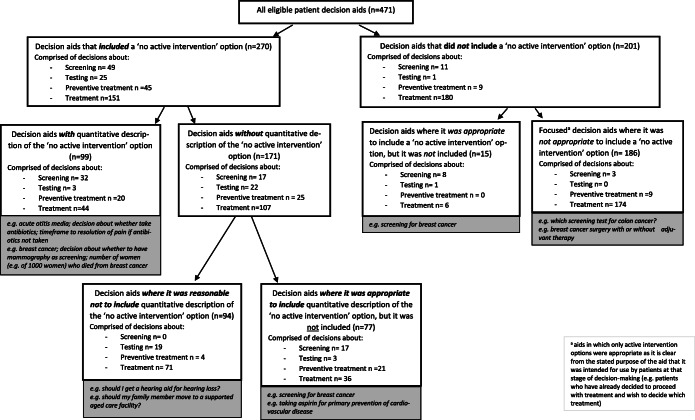
**Decision aids grouped according to whether they included a ‘no active intervention’ option and quantitative description of it.**

We conducted an international environmental scan of decision aids (with no language restriction), searching databases and websites (Box 1) during October 2018. A decision aid was defined as an evidence-based tool designed to help patients make specific and deliberated choices among healthcare options.^5^ To be eligible, the full aid needed to be freely obtainable. Each aid’s content was analyzed independently by two raters. Data extracted included health condition; decision under consideration; whether a ‘no active intervention’ option was presented (if so, the verbatim wording) and whether quantification of that option’s consequences (e.g. likelihood of an outcome, timeframe of illness duration and/or recovery) were provided. For aids without a ‘no active intervention’ option and/or quantitative description, discussion was held between the three authors as to whether that was appropriate. For aids without a ‘no active intervention’ option, we assessed if the aid was a ‘focused’ aid (see Fig. 1) and therefore reasonable to not include this option.

Box 1. Sources Searched to Identify Patient Decision Aids

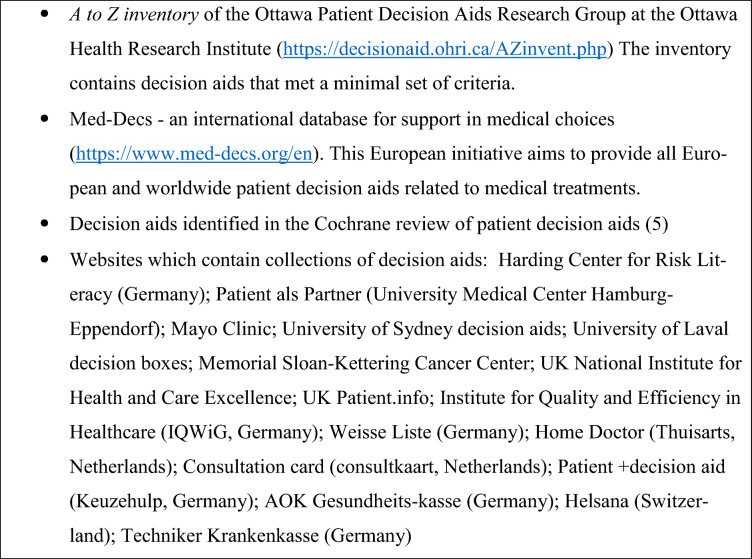



## RESULTS

Of 519 unique decision aids identified, 48 were excluded (5 did not meet decision aid definition; 43 were not accessible), leaving 471 eligible and included Broad decision types addressed were treatment (331, 70%), preventive treatments (except screening) (54, 11%), screening (defined as a test conducted in people without disease signs or symptoms) (60, 13%), and diagnostic tests (26, 6%).

### ‘No Active Intervention’ Option

Just over half (270, 57%) of the aids explicitly included a ‘no active intervention’ option (Fig. 1). Most (206, 76%) worded this as ‘do not do/have…[the active option]’. Other ways used to describe this option were: ‘watchful waiting’ (42, 16%); comparing a treatment to the placebo (aids that were simply converted from a Cochrane review; 10, 4%); self-management of symptoms (7, 3%); and ‘stop taking …[a current treatment]’ (5, 2%).

Of the 201 (43%) aids that did not present a ‘no active intervention’ option, this was appropriate for many (e.g. two methods of childbirth delivery). However, 15 (3%) of the aids could have, but did not, include a ‘no active intervention’ option (see Fig. 1 for examples).

### Quantitative Description of the ‘No Active Intervention’ Option

Of the 270 aids that included a ‘no active intervention’ option, 99 quantitatively described the consequences of that option. We assessed that 77 decision aids could have but did not; and for 94 aids, presenting this information was not reasonable (see Fig. 1 for examples).

## DISCUSSION

Deciding to not have or delay having a screen, test, or treatment is sometimes appropriate. However, this option may be underused unless it is discussed explicitly by clinicians and patients. Unambiguous inclusion of this option in decision aids is important for facilitating such discussions, along with evidence-based estimates of likely timeframes or outcomes if this option is chosen. Our finding that most decision aids included a ‘no active intervention’ option when relevant is reassuring for clinicians and patients. When the option and its outcomes are not provided in aids, clinicians should incorporate this information into the discussion with patients. As it is a criterion in the International Patient Decision Aid Standards,^6^ decision aid developers should ensure this information is included. A possible limitation is that our search may not have located decision aids not contained in any of the major sources searched. Equipping clinicians with the knowledge and skills to have a collaborative discussion with patients when they ask ‘what happens if I do nothing?’ is an important but largely neglected aspect of informed decision-making.
